# The Two-Pore Channel 2 in Human Physiology and Diseases: Functional Characterisation and Pharmacology

**DOI:** 10.3390/ijms26199708

**Published:** 2025-10-06

**Authors:** Laura Lagostena, Velia Minicozzi, Martina Meucci, Antonella Gradogna, Stefan Milenkovic, Fioretta Palombi, Matteo Ceccarelli, Antonio Filippini, Armando Carpaneto

**Affiliations:** 1 Institute of Biophysics, National Research Council, Via De Marini 6, 16149 Genova, Italy; laura.lagostena@ibf.cnr.it (L.L.); antonella.gradogna@ibf.cnr.it (A.G.); 2 INFN and Department of Physics, University of Rome Tor Vergata, Via della Ricerca Scientifica 1, 00133 Roma, Italy; velia.minicozzi@roma2.infn.it; 3 Department of Anatomy, Histology, Forensic Medicine and Orthopaedics, Unit of Histology and Medical Embryology, SAPIENZA University of Rome, 16 Via A. Scarpa, 00161 Roma, Italy; martina.meucci@uniroma1.it (M.M.); fioretta.palombi@uniroma1.it (F.P.); antonio.filippini@uniroma1.it (A.F.); 4 Department of Physics, University of Cagliari, 09042 Monserrato, Italy; stefan.milenkovic@unica.it (S.M.); matteo.ceccarelli@dsf.unica.it (M.C.); 5 Department of Earth, Environment and Life Sciences (DISTAV), University of Genoa, Viale Benedetto XV 5, 16132 Genova, Italy

**Keywords:** TPC channels, ion channels, lysosomes, patch-clamp

## Abstract

Two-pore channel 2 (TPC2) is a member of the endolysosomal ion channel family, playing critical roles in intracellular calcium signaling and endomembrane dynamics. This review provides an in-depth analysis of TPC2, covering its structural and functional properties, physiological roles, and involvement in human diseases. We discuss current experimental approaches to studying TPC2, including heterologous expression in plant vacuoles and computational modeling strategies. Particular emphasis is placed on the structural determinants of ion permeation, with a focus on the selectivity filter and the central cavity’s influence on channel kinetics. Furthermore, we explore emerging roles of TPC2 in viral infections, particularly SARS-CoV-2, and in cancer, including melanoma progression and neoangiogenesis. The inhibitory potential of natural compounds, such as naringenin, is also examined. By offering a comprehensive overview of current knowledge and methodologies, this review underscores the potential of TPC2 as a promising pharmacological target in both infectious and neoplastic diseases.

## 1. The Two-Pore Channel 2

The first functional characterization of TPC channels was carried out in a plant system, specifically in vacuoles isolated from sugar beet roots [1]. Patch-clamp experiments revealed a current that activated over relatively long timescales, generally greater than one-tenth of a second. For this reason, the channel responsible for mediating this current was named the Slow Vacuolar channel, or simply the SV channel. Similar currents, modulated by a variety of parameters, among which are cytosolic calcium and magnesium [2,3], reducing/oxidizing agents [4,5,6,7], polyunsaturated acids [8], and the antibiotic neomycin [9], have been found in vacuoles isolated from all higher plants investigated so far, including those from freshwater [10] and saltwater plants [11]. The model plant *Arabidopsis thaliana* allowed researchers to associate the SV currents with a single gene from the TPC channel family, named AtTPC1 [12].

The functional characteristics of AtTPC1 show marked differences from the human TPC2 (hTPC2) channel: AtTPC1 is an outward rectifier channel, whereas hTPC2 is voltage-independent. AtTPC1 is activated by cytosolic calcium, while hTPC2 is activated by the phosphatidylinositol 3,5-bisphosphate PI(3,5)P_2_, which is ineffective on the plant channel [13]. AtTPC1 is a non-selective cation channel with similar permeability to potassium and sodium; calcium ions can also permeate through the channel [14]; nevertheless, convincing evidence for calcium passing from the vacuolar lumen to the cytosol has never been demonstrated. When activated by PI(3,5)P_2_, the human channel, on the other hand, exhibits specific selectivity for sodium ions, with very low permeability to both potassium and calcium [13,15]. However, hTPC2 was initially discovered as a receptor for nicotinic acid adenine dinucleotide phosphate (NAADP) [16], a potent calcium mobilizer [17], capable of mediating calcium release from the lysosomes to the cytosol when activated by NAADP. In fact, NAADP does not bind directly to the channel but through accessory proteins [18]; when the channel is activated via this pathway, which seems to be followed also by the agonist TPC2-A1-N [19], it behaves as a non-selective cation channel permeable to calcium. This dual nature of hTPC2 provides the channel with considerable flexibility from a physiological perspective [20,21].

## 2. Methods

The hTPC2 channel is located on the lysosomal membrane. Since lysosomes are submicrometric in size, applying the patch-clamp technique is challenging. Several methodologies have been developed, and the advantages and disadvantages of each technique have been discussed in detail in Festa et al., 2022 [22].

### 2.1. Heterologous Expression in Plant Vacuoles

**Figure 1:**
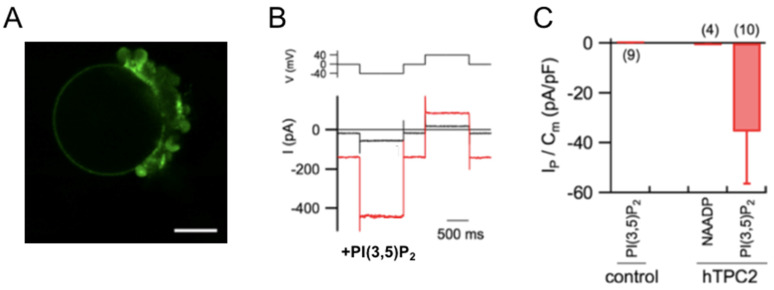
PI(3,5)P_2_, but not NAADP, activates hTPC2 in Arabidopsis vacuoles. (**A**) hTPC2–EGFP localizes to the vacuolar membrane in Arabidopsis mesophyll protoplasts. Shown is a confocal fluorescence image of an isolated vacuole from a protoplast expressing hTPC2–EGFP. (**B**) Representative currents (bottom) from an hTPC2–EGFP–positive vacuole under control conditions (black) and after bath application of 100 nM PI(3,5)P_2_ (red). Voltage protocol shown above. (**C**) Quantification of currents evoked by PI(3,5)P_2_ (330 nM) or NAADP (1 µM) in control and hTPC2–EGFP–expressing vacuoles. Current densities were normalized to membrane capacitance at −40 mV. Numbers in parentheses indicate vacuoles tested. Mean vacuolar capacitance: 25 ± 4 pF. The pipette solution (vacuolar side) contained (in mM): 200 NaCl, 2 MgCl_2_, 10 MES–Tris, pH 5.5. The standard bath (cytoplasmic side) solution contained (in mM): 100 NaCl, 2 MgCl_2_, 10 HEPES–Tris, pH 7.5. The osmotic pressure of the ionic solutions was adjusted to 550 (vacuolar side) and 600 (cytosolic side) mOsm, respectively, by the addition of D-sorbitol. Modification of Figure 2, from Boccaccio et al. [13], reprinted by permission from Springer Nature (license number 6067201350259).

A methodology that has proven very useful for the study of plasma membrane channels and transporters is their expression in heterologous systems. Xenopus oocytes are one of the most commonly used systems [23,24]: these are single cells about 1.0–1.3 mm in diameter, into which the RNA of the membrane protein of interest can be injected. The application of the two-electrode voltage-clamp technique allows for a detailed functional characterization by varying external parameters, as it is very simple to change the solution surrounding the oocyte. If cytosolic parameters are to be studied, the patch-clamp technique in the inside-out configuration is used, employing large-diameter pipettes. Various cell types are also used for heterologous expression. In all these cases, a careful analysis of endogenous channels and transporters is necessary [25,26]. Intracellular channels and transporters can be studied in cells after modifying their targeting through molecular biology. For example, the hTPC2 channel can be redirected to the plasma membrane in HEK293 cells by site-directed mutagenesis Leu11Ala/Leu12Ala at the N-terminal domain [27,28,29]. However, expression in plant vacuoles represents an excellent investigative system [30,31,32]. The plant vacuole is an internal compartment within the plant cell that can occupy up to 90% of the cellular volume in mature cells. It is easy to isolate, and the patch-clamp technique can be applied in all configurations. Additionally, the cytosolic side is exposed outward, allowing for easy study of cytosolic parameters. In Figure 1, we observe how hTPC2, fused to GFP, is localized on the tonoplast (the vacuolar membrane) and how PI(3,5)P_2_ is capable of evoking currents, while NAADP, in the absence of the mediator protein, does not generate significant currents.

**Figure 2:**
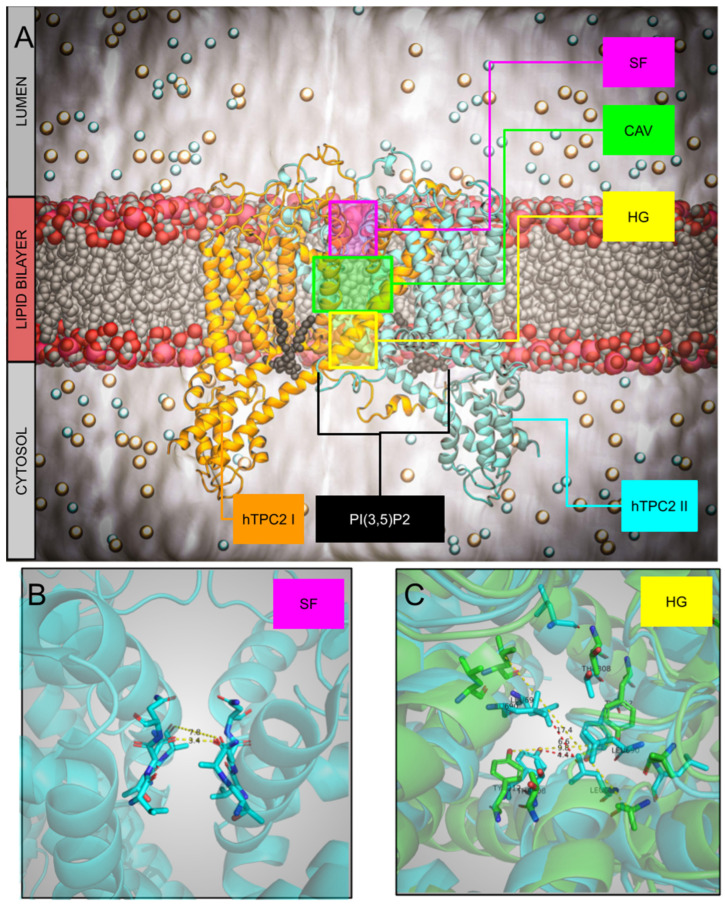
(**A**) Overview of the hTPC2 system with PI(3,5)P2 depicted as black spheres and marked selectivity filter (SF), central cavity (CAV), and hydrophobic gate (HG). (**B**) The selectivity filter of the hTPC2 with T271, A272, V652, N653, and N654. (**C**) Hydrophobic gate of the apo closed (cyan, pdbid 6NQ1) and holo open (green, pdbid 6NQ0) with residues T308, Y312, L690, and L694.

### 2.2. Computational Methods

Complementing and bridging advances in experimental methods, computational methods have proven very useful in the last decade for studying ion-channels [33]. In particular applying classical molecular dynamics (MD), in its standard form or with several biases (external constant electric field, multiple replicas with increasing temperatures [34], umbrella sampling and eventually metadynamics), allowed to investigate in details both the structure of TPC2 and its mechanism of ions permeation. Thanks to MD simulations, we shed light on the mechanism of ligand-gating [35] and the role of central cavity (CAV) in sodium transport [36], and eventually on the conformational flexibility of the selectivity filter (SF) [37,38].

When coupled with Markov state models [39], MD simulations offer a unique multi-scale insight into sodium transport kinetics [36]. Another perspective of TPCs’ structure-function relation could be pursued through investigation of ligand interactions and identification of potential small-molecules inhibitors/modulators. This kind of investigation is usually conducted via docking which can help to identify the binding site of naringenin, an efficient inhibitor of hTPC2 [40,41]. Another potent method in quantifying function-affecting potency of a small molecule is cross-correlation matrix that was used numerously for detecting and describing signals small molecule produce when bound to protein [35,36,42,43,44]. Finally, it is worth mentioning that very recently Machine Learning (ML) techniques proved its value for predicting microbiological properties of small molecules particularly when coupled with MD-based statistics [45] offering a promising approach in future identification of TPCs’ inhibitors.

## 3. Structure

TPC channels are one of the eight families that belong to the voltage-gated ion channel superfamily [46]. The hTPC2 channel consists of 752 amino acids (see UniProt accession number: Q8NHX9, or NCBI accession: AY029200.1) and is composed of two homologous Shaker-like subunits linked by a cytosolic linker, each containing six transmembrane segments IS1-S6 and IIS1-S6 (or in some studies, S1–S6 and S7–S12). In both subunits, between S5 and S6, there is a hairpin structure called the P-loop (P for pore), which contributes to the formation of the channel’s selectivity filter. Four pore-loops are required to form a channel, so the structure of hTPC2 is homodimeric; it is positioned between the tetrameric structure of potassium channels, which are formed by four non-covalently linked Shaker-like subunits, and the monomeric structure of sodium and calcium channels, in which Shaker-like subunits are covalently linked [47]. Consequently, a single mutation in the protein sequence of hTPC2 results in a double mutation in the functional channel. Additionally, the channel has a single permeation pore, contrary to what the name suggests, and should more appropriately be called a Two Pore-loop forming Domain Channel (TPDC). While S5 and S6, through the P-loop, form the permeation pore, the S1–S4 subunits are referred to as the Voltage-Sensing Domain (VSD) because, with the charged amino acids, typically arginines, present on the S4 helix, they generally form the part of the channel capable of sensing changes in membrane potential.

Through a combined approach of computer simulations and mutagenesis experiments, performed by expressing the mutated channel in vacuoles from *Arabidopsis thaliana* mesophyll cells lacking the endogenous AtTPC1, Kirsch et al. [48] identified the binding site of PI(3,5)P_2_. The phosphoinositide interacts with the first subunit of the channel, particularly with the positively charged amino acids K203, K204, and K207 located in the linker between the S4 and S5 transmembrane helices, and with S322, which is found in the cytosolic extension of S6 [48]. These findings were confirmed by the structures of hTPC2 obtained through cryo-EM. Three different conformations of the channel were observed: the apo closed conformation (non-conductive), the ligand-bound closed conformation (also non-conductive), and the ligand-bound open conformation (conductive). When PI(3,5)P_2_ is bound, the closed and open channel states are in equilibrium in a 3:5 ratio [29]. The non-conductive state is due to the interaction between the S6 helices, mediated by four pairs of residues: Thr308 and Tyr312 from IS6, and Leu690 and Leu694 from IIS6. This interaction creates a constriction on the cytosolic side, which prevents the passage of hydrated cations. The binding of PI(3,5)P_2_ does not induce a significant conformational change in the channel; the apo closed and ligand-bound closed states are essentially identical, except for the presence of the phosphoinositide. However, the phosphate group of PI(3,5)P_2_ interacts with Asp329 on IS6, inducing a conformational change in IS6 that is transmitted to IIS6. Overall, these movements of the S6 helices open the channel. In hTPC2, the opening mechanism is not voltage-dependent.

As discussed by She et al. [29], although the VSD (Voltage Sensing Domain) contains the IS4 helix, which has some positively charged amino acids (arginines), it has several features that render it insensitive to voltage: in S3, a basic residue involved in the gating charge is absent, and in IS4, a positively charged arginine typically found in voltage-gated channels is replaced by phenylalanine (Phe194). Additionally, IS4 forms a regular alpha-helix instead of a 3_10_-helix. Regarding the second VSD, one amino acid is essential for voltage-dependence. The mutation of isoleucine at position 551 with an arginine converts hTPC2 into a voltage-dependent channel, similar to what occurs in MnTPC1, where arginine is found at the corresponding position instead of isoleucine [29].

It is interesting to note that high-throughput screening experiments followed by electrophysiology applied to enlarged lysosomes identified some agonists belonging to the class of tricyclic antidepressants (TCAs) that can confer voltage dependence to the channel [49]: unlike hTPC1, which acts as an outward rectifier [50], hTPC2 behaves as an inward rectifier. Interestingly, the application of the flavonoid naringenin inhibits hTPC2 currents but enhances the currents evoked by desipramine (one of the TCAs found to be an agonist by Zhang et al. [49]). These results can be explained by the presence of an intermediate state of IIS4 (also found in TPC3 of *Xenopus tropicalis*), on which desipramine acts in a voltage-dependent manner. Docking experiments of naringenin on the various structures of hTPC2 [41] show that, due to its small size and hydrophobicity, it can bind to multiple sites on the channel. However, the site that induces the inhibition of currents activated by PI(3,5)P_2_ remains elusive.

Tetrandrine, an alkaloid extracted from the plant *Stephania tetrandra*, is an inhibitor of TPC2 [51]; recently, Chi et al. [52] demonstrated that SG-094, a synthetic analog of tetrandrine, can inhibit TPC2 by locking the IIS4 helix in a conformation that prevents channel opening.

### The Mechanism of Ions Permeation: The SF and the Role of Central Cavity on Kinetics

Though formed by two pores, as reported in Figure 2 (Panel A, colored orange and cyan), TPCs share with other ion channels the three typical structural elements, (i) a selectivity filter (SF, panel B), (ii) a water-filled central cavity (CAV, highlighted in green, panel A), and (iii) a hydrophobic gate (HG, panel C) [53,54]. For the latter, we superimposed the apo closed (cyan, pdbid: 6NQ1) and holo open (green, pdbid: 6NQ0) states, with their main distances. MD simulations with their time and space resolution power represent a key tool to investigate the role of these elements and the mechanism of the flow of ions [55].

Two recent and concomitant papers reported a new state of the TPC2’s SF that has not been captured by the various cryo-EM structures appeared in recent years [37,38]. Starting from the PI(3,5)P_2_ bound structure (6NQ0) MD simulations revealed an expansion of the filter II that permits the permeation of both Na^+^ and Ca^2+^. It can be speculated that this “super-open” state observed also in [38] is reached when the channel is activated by NAADP through accessory proteins [18,19]. To note the in both [37,38] these new states came as results of MD simulations, pointing to a high degree of flexibility of the SF and HG regions. This is not surprising as the structures of both TPC1 and TPC2 showed an intrinsic flexibility in the presence of PI(3,5)P_2_. The principal component analysis (PCA) pointed out an anticorrelated movement of the two monomers allowing the opening of the hydrophobic gate, probably concomitant with the expansion of the SF [38]. Speculatively, this characteristic might be assigned to the specificity of the dimeric form of TPCs. Further, the presence of a disulfide bonds at the luminal mouth seems to avoid the collapse of the long loops, thus permitting the entry of ions in the SF [38].

The central cavity, another crucial structural feature of ion channels, with its position in the middle of the membrane and filled with water molecules, facilitates ions to cross the hydrophobic environment. A multiscale analysis, combining MD simulations, enhanced MD simulations, and an analytical-numerical approach, was used to understand the particular mechanism in TPC, with ions diffusing slowly and/or fast, with a knock-off mechanism [36]. The comprehensive methodology allowed for a detailed exploration of the interactions between sodium ions and the channel’s structural components. The diffusion of a single sodium ion was quantified via free energy calculations when the cavity is occupied by none, one or two additional sodium ions, revealing different profiles. These three conditions mimic an increased external concentration. Because of the high steric barrier represented by the hydrophobic gate, the cavity works as a reservoir for sodium ions. At low concentration the probability to occupy the cavity and the residence time of the single ion are both very high, thus reducing the flow of ions. On the other hand, at medium and high concentration, when the cavity is presumably occupied by one or two sodium ions, the residence time of sodium is lower than before, allowing a larger flow. Thus, the presence of the cavity with multiple occupancy modulates the flow of ions at increasing concentrations, allowing a linear regime of conductance up to mM concentration of sodium ions. The interactions within this cavity are essential for maintaining the channel’s function, and disruptions could lead to impaired ion flow. Further, the more recent simulations with a large SF that permitted a better sampling of permeation events confirmed the presence of a binding site for sodium inside the cavity [38]. This binding site promotes a knock-off mechanism (or distant knock-on), as already suggested in [36].

In summary, TPC has the same elements as other ion channels: SF, cavity, HG. However MD simulations revealed specific behaviors: (i) the plasticity of SF upon sodium binding (ref. [38] reported that PI(3,5)P_2_ interacts with the VSD regions, not active here), and (ii) the particularity of the cavity, that can be occupied by more than one ion, (iii) the high barrier at the HG. Further, MD simulations suggested the existence of a super-open or real-open state, where an expansion of the SF is seen. Thus, the structure 6NQ0, for which a low conductivity was calculated with MD simulations in agreement with experimental data [36] might represent a state highly selective for sodium and with low conductivity, in contrast to the new state that can transport both calcium and sodium.

## 4. Role of TPC2 in Physiology and Human Diseases

Lysosomes are no longer considered just the cell’s waste disposal system but are involved in crucial cellular functions such as secretion, autophagy, cargo sorting, plasma membrane repair, signaling, energy metabolism, and degradation; consequently, lysosomal malfunction has been associated with many human diseases [56]. TPC2 is expressed in late endosomes and lysosomes (LELs, late endosomes/lysosomes) and is therefore important in lysosomal physiology [20]. The dual nature of the channel can lead to different behaviors [57]: when activated by PI(3,5)P_2_, it should induce significant depolarization of the lysosomal membrane, with possible effects on fusion mechanisms; it could also result in the release of osmolytes from the lysosomal lumen [58]. It can also regulate endomembrane tension to enable remodeling and resolution of phagolysosomes [59]. When activated by NAADP, TPC2 would lead to a localized increase in cytosolic Ca^2+^, which, through a Ca^2+^-induced Ca^2+^ release mechanism, amplified by IP_3_R receptors, could result in a global increase in Ca^2+^; furthermore, PI(3,5)P_2_ and NAADP may act synergistically [60]. TPC2 plays a pivotal role in endo-lysosomal functions, including trafficking, autophagy, exocytosis, and maintaining lysosomal cation and pH homeostasis [61,62,63,64].

**Figure 3:**
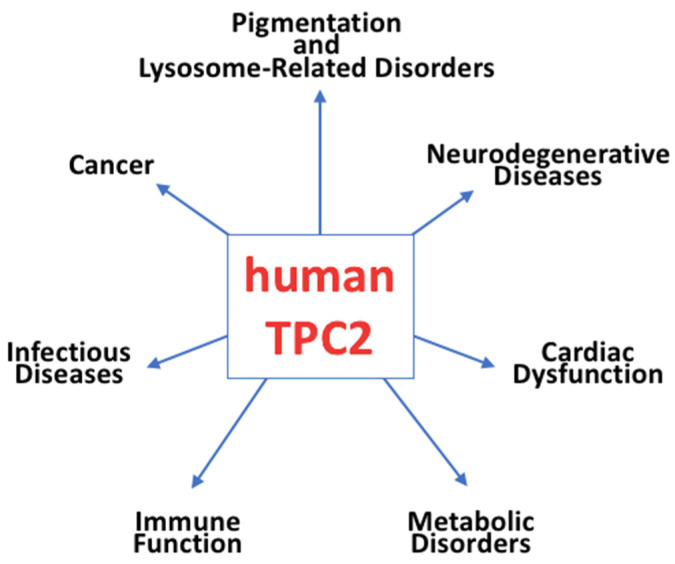
Schematic representation of TPC2 as an emerging ion channel implicated in various diseases.

TPC2 has recently been recognized as a critical intracellular ion channel with profound physiological and pathophysiological significance [57,64,65]. Emerging research highlights its involvement in various processes and diseases, shown in Figure 3:

**Pigmentation and Lysosome-Related Disorders**: Variations in TPC2 activity have been associated with pigmentation differences and hair color variation.

**Neurodegenerative Diseases**: Alterations in TPC2 function disrupt lysosomal homeostasis, contributing to the pathogenesis of neurodegenerative disorders.

**Cardiac Dysfunction:** TPC2 has been implicated in the pathophysiology of cardiac dysfunction, particularly in heart failure and hypertrophic signalling.

**Metabolic Disorders**: Dysregulated TPC2 activity has been linked to metabolic conditions, including obesity and diabetes, through its impact on lysosomal signaling and nutrient sensing.

**Immune Function**: TPC2 affects immune responses by regulating macropinocytosis and phagocytosis in macrophages, essential for pathogen clearance and immune surveillance.

**Infectious Diseases**: TPC2 plays vital roles in viral infections, including those caused by Ebola virus, HIV-1, and SARS-CoV-2, by modulating viral trafficking and degradation within endolysosomal compartments.

**Cancer**: TPC2 has been identified as a key regulator in cancer progression, influencing cell migration, metastasis, and angiogenesis.

### 4.1. Pigmentation and Lysosome-Related Disorders

TPC2 has emerged as a critical player in the regulation of hair pigmentation, with its activity directly influencing melanin production and pigmentation phenotypes. Variants of TPC2 have been linked to hair color variation through gain-of-function (GOF) mechanisms that alter channel activity [66]. Notably, the M484L variant increases sensitivity to the endogenous ligand PI(3,5)P_2_, enhancing channel activity, while the G734E variant reduces inhibition by ATP, further contributing to altered pigmentation. These findings highlight the role of TPC2 in pigmentation defects and hair color diversity. Moreover, the L564P variant, identified as the predominant polymorphism in human populations, has been shown to act as a prerequisite for the blond hair-associated GOF effect of M484L, emphasizing the complex genetic interplay underlying TPC2-mediated pigmentation [64]. Together, these findings underscore the intricate role of TPC2 in modulating hair color and its genetic underpinnings.

TPCs play pivotal roles in the function and dynamics of lysosome-related organelles (LROs), including platelet dense granules, melanosomes, and cytolytic granules [64]. TPC2, in particular, has been implicated in regulating membrane dynamics and Ca^2+^ signaling, critical processes for LRO function. For example, TPC2-mediated Ca^2+^ release governs the membrane dynamics of platelet dense granules, influencing platelet function and aggregation [67]. In melanosomes, TPC2 regulates pigmentation by modulating melanosome pH and size, essential for proper melanin production and storage [68]. Additionally, Na^+^ ion transport through melanosomal TPCs contributes to pigmentation regulation, highlighting their role in ion homeostasis within LROs [69]. Beyond pigmentation, TPC2 facilitates calcium release from T-cell cytolytic granules, promoting exocytosis and target cell killing, thereby linking TPC2 function to immune responses [70]. These findings underscore TPC2’s broad impact on LRO-related pathologies and physiological processes.

### 4.2. Neurodegenerative Diseases

TPC2 in neurodegenerative processes is an emerging area of research, highlighting its role in lysosomal function and cellular homeostasis. Emerging evidence highpoints the pivotal role of lysosomal dysfunction in neurodegenerative diseases, including Parkinson’s disease (PD), Alzheimer’s disease (AD), Huntington’s disease (HD), amyotrophic lateral sclerosis (ALS), and lysosomal storage disorders (LSD) [71]. Central to this dysfunction is impaired autophagic flux, which results in the accumulation of toxic aggregates and defective clearance of damaged organelles. TPCs, particularly TPC2, have been identified as critical regulators of lysosomal ionic homeostasis and autophagy.

Research by Hockey et al. [72] demonstrated that TPC2 inhibition could counteract lysosomal morphological abnormalities induced by pathogenic LRRK2 in PD models, providing a potential therapeutic approach for restoring lysosomal function. A new study reveals that lysosomal TPC2 channels are disrupted by the common LRRK2 G2019S mutation, a cause of PD, by causing excessive Ca^2+^ entry into dopaminergic neurons. This disruption perturbs normal Ca^2+^ homeostasis and dopamine-dependent functions, and can be corrected by reducing the Ca^2+^ permeability of TPC2 channels, suggesting a potential new therapeutic target for Parkinson’s Disease [73]. This research identifies the lysosomal TPC2 channel as a novel and potentially druggable target for LRRK2-linked Parkinson’s disease. By understanding the mechanisms by which LRRK2 mutations affect lysosomal calcium signaling through TPC2, new therapeutic strategies can be developed to restore proper calcium homeostasis and protect dopaminergic neurons. Further, Tedeschi et al. [71] underscored the therapeutic potential of targeting lysosomal channels like TPC2 and TRPML1 to modulate autophagic processes, highlighting their role in ameliorating neurodegenerative phenotypes. In HD, it has been shown that NAADP-evoked Ca^2+^ signaling through TPCs exacerbates mutant huntingtin aggregation and autophagy impairment, further establishing the relevance of TPCs in disease pathophysiology [74].

Interestingly, recent studies have shown that TPCs influence not only cellular homeostasis but also systemic functions, such as oxytocin secretion and social behavior [75]. This study reveals a surprising role of lysosomes and TPCs in the regulation of neuropeptide secretion and their influence on social behavior.

Beyond specific diseases, TPC2 contributes broadly to lysosomal integrity in LSDs, where its modulation has shown potential to rescue lysosomal dysfunction by restoring ion balance and autophagic processes [76]. The ability of TPC modulation to affect such diverse processes underscores their broad therapeutic potential. However, despite the identification of some pharmacological modulators for TPCs, more specific and effective agents are needed to mitigate autophagy dysfunction across neurodegenerative conditions [71].

In summary, targeting TPC2 and other lysosomal channels may offer novel pharmacological strategies to enhance autophagy and mitigate neurodegenerative processes, positioning TPC2 as a promising molecular target in therapeutic interventions.

### 4.3. Cardiac Dysfunction

TPCs have been implicated in ischemia reperfusion injury, arrhythmias and cardiac hypertrophy [65].

Elevated levels of TPC1 and TPC2 are reported in left ventricular samples from patients with ischemic and dilated cardiomyopathy, suggesting a correlation between TPC expression and heart failure [77]. Moreover, increased expression of genes associated with fatty acid and calcium metabolism were identified in failing human hearts, linking TPC2’s role in intracellular ion dynamics to metabolic dysregulation in cardiac tissue [77]. Furthermore, de Zélicourt et al. [78] highlighted TPC2 as a central hub in excitation-contraction coupling and hypertrophy signaling. This positions TPC2 as a critical regulator of Ca^2+^ handling, energy metabolism, and stress responses in cardiomyocytes. Collectively, these findings suggest that dysregulated TPC2 activity may contribute to the progression of cardiac dysfunction, offering potential therapeutic avenues for treating heart failure and related conditions.

### 4.4. Metabolic Disorders

TPC2 plays a multifaceted role in glucose homeostasis, influencing pancreatic cell function and systemic glucose regulation. Arredouani et al. [79] demonstrated that TPC2, activated by NAADP, modulates Ca^2+^ dynamics and contributes to membrane excitability and stimulus-secretion coupling in pancreatic β-cells. This suggests TPC2’s involvement in insulin secretion, a critical component of glucose regulation. Subsequent research revealed that TPC2 is dispensable for normal Ca^2+^ signalling and insulin secretion in β-cells, indicating that its role might vary depending on physiological conditions [80]. Beyond β-cells, Hamilton et al. [81] showed that TPC2-dependent Ca^2+^ mobilization in pancreatic α-cells mediates adrenaline-induced glucagon secretion. These findings suggest that TPC2 activity impacts both insulin and glucagon secretion, positioning it as a significant regulator of glucose homeostasis and a potential target for diabetes therapy.

TPCs have emerged as critical regulators of lipid metabolism and energy homeostasis, with significant implications for metabolic diseases. TPC2, in particular, has been identified as a key player in liver and adipose tissue physiology. TPC2-deficient mice are highly susceptible to fatty liver disease, highlighting TPC2’s role in lipid processing and hepatocyte function [82]. The study underscored the importance of intracellular ion channels in maintaining lipid balance and preventing steatosis. Similarly, Lear et al. [83] revealed that the absence of both TPC1 and TPC2 leads to mature-onset obesity in male mice. This phenotype is linked to impaired lipid mobilization for thermogenesis in brown adipose tissue, emphasizing the role of TPCs in energy expenditure. Together, these findings position TPC2 as a potential therapeutic target for addressing metabolic disorders such as fatty liver disease and obesity.

### 4.5. Immune Function

TPCs are suggested to play critical roles in immune cell function, such as macro-pinocytosis and phagocytosis in macrophages [84]. In particular, TPC2 has emerged as a pivotal regulator in the immune system, influencing processes such as endocytosis, phagocytosis, and macropinosome resolution. Freeman et al. [58] demonstrated that lipid-gated ion fluxes through TPC2 are essential for regulating endocytic trafficking and immune surveillance, emphasizing the channel’s role in cellular processes critical to immune function. Similarly, it has been demonstrated that TPC2-mediated Ca^2+^ nanodomains, triggered by NAADP, drive phagocytosis in immune cells by activating calcineurin and dynamin [85]. Further research supported TPC2’s involvement in resolving macropinosomes, a process integral to antigen processing and presentation [86]. Additionally, TPC2 was implied in cancer immunity, suggesting its influence on endolysosomal trafficking could modulate immune responses in oncological contexts [87]. A comprehensive overview of TPC2’s roles across the immune system is provided in Steiner et al. [88], reinforcing its significance in both innate and adaptive immunity. These findings underscore TPC2 as a critical player in immune regulation and a potential target for therapeutic interventions in immune-related disorders.

### 4.6. Viral Infections

TPC2 is also involved in viral infections, where it plays a pivotal role in endosomal trafficking and virus–host interactions. Many viruses exploit host cellular pathways for their entry and replication. As a regulator of endosomal pH and Ca^2+^ release, TPC2 is a key player in these processes. Its critical roles have been demonstrated in infections caused by viruses such as Ebola [51], coronaviruses [89], HIV-1 and SARS-CoV-2 [90]. By modulating viral trafficking through endo-lysosomal pathways, TPC2 emerges as a promising target for therapeutic intervention.

#### SARS-CoV-2 Infection

The role of TPC2 in SARS-CoV-2 infection garnered significant attention during the COVID-19 pandemic. SARS-CoV-2 uses the endolysosomal system for entry into host cells [90], a process that depends on the acidification of endosomes and the activity of lysosomal proteases. Studies have demonstrated that pharmacological inhibition of TPC2 reduces viral entry and replication in vitro. By modulating endosomal calcium release and pH homeostasis, TPC2 inhibitors could interfere with the viral life cycle.

Recently, Naringenin, a natural flavonoid, has garnered attention for its potential therapeutic effects against SARS-CoV-2 infection [91,92,93] and has been proposed as a potential treatment for long COVID disease [94]. Its multifaceted mechanism of action, including antiviral, anti-inflammatory, and antioxidant properties, suggests that it could complement existing antiviral therapies or serve as a preventive measure for high-risk populations. Preclinical studies [92] and in silico analyses [95,96] support these potential benefits, but more robust clinical trials are necessary to confirm its efficacy and safety in treating COVID-19. As a dietary supplement, naringenin could complement existing therapeutic strategies, though it should not replace established medical treatments for the infection. Further studies are needed to establish its efficacy, optimal dosing, and potential interactions in the context of SARS-CoV-2 infection.

### 4.7. Cancer

Cancer is a complex multifactorial disease. According to the World Health Organization (WHO), it is a major global public health challenge and the second leading cause of death. It is characterized by the uncontrolled proliferation of cells that invade surrounding tissues and spread to distant organs. This aberrant growth is caused by genetic mutations and epigenetic changes, which interfere with cellular control mechanisms and allow malignant transformation; sustained cell proliferation, evasion of growth suppressors, resistance to apoptosis, and neo-angiogenesis are key hallmarks of cancer [97]. Over the years, multiple therapeutic approaches have been developed to fight cancer, from surgical removal, chemotherapy and radiotherapy to more advanced treatments like immunotherapy and targeted therapy. However, a major challenge in oncology is the tendency of tumours to progressively develop resistance to these treatments [98]. Consequently, there is a continuous need to identify and develop new therapeutic strategies that can complement and enhance the effectiveness of existing therapies. Calcium is a ubiquitous secondary messenger and Ca^2+^ signalling plays a central role in cancer progression, from regulating cell proliferation and evading apoptosis to promoting metastasis and drug resistance, making it a complex but promising target for therapeutic intervention [99,100]. To this purpose, findings describing the emerging role of lysosomal Ca^2+^ channels such as TPCs has been recently reviewed [101].

TPCs, particularly TPC2, are central to calcium (Ca^2+^) signaling within endolysosomal compartments, playing a significant role in cancer progression [102]. TPC2-mediated Ca^2+^ release, triggered by nicotinic acid adenine dinucleotide phosphate (NAADP), supports key oncogenic processes such as cell migration, invasion, and angiogenesis. TPC2 plays an essential role in the migration of invasive cancer cells, suggesting its potential as a therapeutic target [103]. Similarly, Favia et al. [104] demonstrated that VEGF-induced angiogenesis—a critical component of tumor growth—is mediated by TPC2-dependent Ca^2+^ signaling. Beyond these findings, Parrington et al. [105] emphasized the broader impact of TPC2 in regulating developmental and pathological processes, including cancer. Recent studies also revealed therapeutic avenues targeting TPC2. For example, the TPC antagonist tetrandrine was shown to block autophagy, enhancing the cytotoxic effects of sorafenib in hepatocellular carcinoma cells [106]. Collectively, these studies underscore TPC2’s pivotal role in cancer biology and its potential as a novel target for therapeutic interventions [107].

#### 4.7.1. Melanoma

Malignant melanoma (MM) is one of the most aggressive and treatment-resistant types of cancer. It is a complex and heterogeneous disease with genetic, clinical, and histopathological variations responsible for unpredictable treatment effectiveness [108]. Melanoma is caused by the uncontrolled proliferation of melanocytes, cells based on the basal layer of the epidermis, which accumulate genetic defects. This leads to the formation of an in situ lesion, characterized by its ability to grow horizontally within the epidermis. As the disease progresses, tumour cells acquire the capability to grow vertically and start to invade the dermis and subcutaneous tissue and to metastasize [109].

Melanocytes are characterized by the ability to produce the pigment melanin, which is synthesized and stored in intracellular organelles (melanosomes) before being transferred to adjiacent keratinocytes as a protection against UV-induced cell damage. Melanosomes are lysosome related organelles (LROs), sharing several characteristics with this family of acidic compartments. One of these is the expression of the TPC2 on their membrane, which is necessary for melanosomal physiology, including pH regulation and melanin production [68,110].

Given that TPC2 regulates the homeostasis of calcium (Ca^2+^) and sodium (Na^+^), it has attracted considerable interest from a pathological standpoint, particularly in the context of tumorigenesis not only in melanoma but also in various other cancer types. Indeed, as shown previously, it has been demonstrated that both genetic and pharmacological inhibition (via siRNA or via the inhibitors NED-19 and Tetrandrine) significantly reduce the migration of urinary bladder and hepatic human carcinoma cells and murine breast cancer cells through the dysregulation of the β1-integrin signalling pathway in in vitro studies [103]. Moreover, in vivo studies have shown that the selective TPC2 inhibitor, SG-094, effectively impairs cell proliferation and tumour growth of hepatocellular carcinoma [111]. Data from these models suggest a pro-tumoral role for TPC2.

Surprisingly, data in malignant melanoma question this simplistic deduction. Unlike in other cancer types, studies indicate that the loss of TPC2 expression actually enhances metastatic traits in CHL1 (BRAFw.t.) human melanoma metastatic cell lines [112], as well as in B16 F10 murine melanoma cells [113]. In contrast, in other studies it has been shown that flavonoids can increase melanin production in MNT-1 (BRAF V600E) human melanoma metastatic cells, while reducing cell proliferation, migration and invasion by inhibiting the TPC2 channel [114]. Specifically, D’Amore et al. [112] demonstrated that TPC2 Knockout (ko) in CHL1 cells increases invasiveness and alters the expression of epithelial–mesenchymal transition (EMT) markers, suggesting a shift towards a more aggressive phenotype. This transformation is mediated by a mechanism involving Hippo pathway signals (YAP/TAZ) and Store-Operated Calcium Entry (SOCE). On the other hand, Netcharoensirisuk et al. [114] showed that the inhibition of TPC2 reduces the levels of MITF, a transcription factor that promotes melanoma progression, while increasing the activity of tyrosinase, an enzyme essential for melanin synthesis. The researchers also identified specific flavonoids, such as MT-8 and UM-9, that effectively block TPC2, boosting melanin content and reducing melanoma cell aggressiveness. At the molecular level, a very recent study [115] has shown how Rab7a, a small GTPase, physically controls the activity of TPC2, which modulates the GSK3β/β-Catenin/MITF axis. In particular, Abrahamian et al. [115] have demonstrated in in vitro and in vivo studies how the knockout of either Rab7a or TPC2 lead to a less aggressive phenotype in MITF-high human melanoma cell lines. This results in a decrease in the level of this protein which is degraded by the proteasome system after being phosphorylated by the GSK3β kinase. This process also involves the degradation of β-Catenin, a known regulator of melanoma cell growth that has MITF as a downstream target. These findings underscore that the role of TPC2 is highly dependent not only on the type of tumour analysed but also, likely, on the genetic background. Specifically, the distinct function of TPC2 observed in the two human metastatic melanoma cell lines, CHL-1 and MNT-1, may be attributed to the presence of the BRAF mutation, which is found in 50% of all MM cases. Further research in melanoma emphasizes that disease stage is also a critical factor, as TPC2 shows distinct roles in primary versus metastatic melanoma. For instance, Barbonari et al. [113] observed that in primary murine melanoma cells (B16F0), TPC2 knockdown reduces cell migration and invasion, suggesting a role in promoting tumour growth and spread. Conversely, in the metastatic subline of the same murine melanoma (B16F10), TPC2 knockdown or knockout paradoxically increases cell migration and invasion, with a concomitant increase in EMT markers. This implies that, in more advanced cancer stages, TPC2 may exert an anti-metastatic effect. This unexpected dual role suggests that TPC2 functions as a context-dependent regulator of melanoma progression, exhibiting opposing effects based on the tumour’s stage. These studies were primarily conducted in vitro; however, in vivo studies have provided further insights. Notably, TPC2 knockout significantly reduces the ability of melanoma-derived cells to grow in vivo but paradoxically leads to increased systemic toxicity, particularly in liver, spleen, and lung tissues [116]. Intriguingly, the plant alkaloid tetrandrine was recently shown to enhance melanoma cell immunogenicity by simultaneously blocking autophagic flux and proteasomal activity, thereby promoting MHC-I presentation [117]. Given the established role of TPC2 in controlling lysosomal function, it is tempting to speculate that some of the immunomodulatory effects of tetrandrine could be mediated, at least in part, through interference with TPC2-dependent pathways. These findings identify TPC2 as a promising yet complex therapeutic target whose effects vary not only by cancer type but also by disease stage. This highlights the importance of precision oncology approaches that align treatment with tumour biology. Further research into TPC2’s mechanisms could deepen our understanding of melanoma progression and inform treatment strategies, especially in metastatic stages where modulating TPC2 may influence pathways associated with cancer aggressiveness.

#### 4.7.2. Neoangiogenesis and Naringenin Inhibition

Neoangiogenesis, the formation of new blood vessels from pre-existing ones, is a critical process in both physiological and pathological contexts, such as tissue repair, cancer progression, and ischemic diseases. Neoangiogenesis is strongly regulated by intracellular Ca^2+^ signaling [118]. A recent study has highlighted the significant role of TPC2 in regulating this process. Specifically, TPC2 mediates angiogenic responses through the potent Ca^2+^-mobilizing messenger NAADP, which triggers the release of intracellular Ca^2+^ from TPC2-associated stores [104].

Naringenin, a natural flavonoid abundantly found in citrus fruits, has garnered significant attention as a promising inhibitor of TPC2-mediated pathways [40,41]. Extensive studies have demonstrated its potential as a novel pharmacological agent, exhibiting enhanced potency, specificity, and favorable drug-like properties, which position it as a viable candidate for therapeutic applications [114,119,120,121].

Emerging evidence reveals that naringenin effectively suppresses neoangiogenesis by targeting and disrupting TPC2-mediated Ca^2+^ signaling pathways. In addition, naringenin has been reported to activate M-type K^+^ channels and large-conductance Ca^2+^-activated K^+^ channels, which may further contribute to its vascular effects [122]. Specifically, naringenin inhibits Ca^2+^ influx, a critical step that regulates key cellular processes involved in angiogenesis, including endothelial cell proliferation, migration, and tube formation [121]. These processes are indispensable for the development of new blood vessels, which play a central role in both physiological and pathological conditions. By impairing these essential cellular mechanisms, naringenin demonstrates its ability to interfere with aberrant angiogenesis [121]—a hallmark of diseases such as cancer, diabetic retinopathy, and other ischemic disorders. Its multi-faceted inhibitory effects underscore its potential as a therapeutic agent capable of modulating pathological angiogenesis, offering a targeted approach with minimal off-target effects. The growing body of evidence supporting naringenin’s mechanism of action further strengthens its candidacy as a safe and effective treatment option in diseases characterized by excessive or dysregulated blood vessel formation.

## 5. Conclusions

TPC2 emerges as a multifaceted ion channel with pivotal roles in regulating intracellular signaling pathways and maintaining endomembrane homeostasis. Its complex structure, particularly the selectivity filter and central cavity, underlies a finely tuned ion permeation mechanism that is essential for its physiological function. Advances in experimental and computational approaches have significantly deepened our understanding of TPC2, enabling detailed characterization of its activity and pharmacological modulation. Importantly, the channel’s implication in pathophysiological contexts such as viral infections—most notably SARS-CoV-2—and cancer progression, including melanoma and neoangiogenesis, underscores its clinical relevance. The identification of natural inhibitors like naringenin further opens avenues for therapeutic intervention. Collectively, these insights position TPC2 as a promising and versatile target in the development of new strategies for the treatment of infectious and oncological diseases.

## Data Availability

No new data were generated or analyzed in this study.

## Abbreviations

AD	Alzheimer’s Disease
ALS	amyotrophic lateral sclerosis
CAV	Central Cavity
EGFP	Enhanced Green Fluorescent Protein
EMT	epithelial–mesenchymal transition
GFP	Green Fluorescent Protein
HD	Huntington’s disease
HG	Hydrophobic Gate
LEL	Late Endosomes/Lysosomes
LRO	Lysosome-Related Organelle
LSD	lysosomal storage disorders
MD	Molecular Dynamics
ML	Machine Learning
NAADP	nicotinic acid adenine dinucleotide phosphate
PD	Parkinson’s Disease
PI(3,5)P_2_	phosphatidylinositol 3,5-bisphosphate
SF	Selectivity Filter
SOCE	Store-Operated Calcium Entry
TPC2	Two Pore Channel 2
VSD	Voltage Sensing Domain
